# Comparison of vascular parameters between normal cynomolgus macaques and healthy humans by optical coherence tomography angiography

**DOI:** 10.1186/s12886-019-1207-x

**Published:** 2019-10-11

**Authors:** Jingyi Peng, Liuxueying Zhong, Li Ma, Jiayi Jin, Yongxin Zheng, Chenjin Jin

**Affiliations:** State Key Laboratory of Ophthalmology, Zhongshan Ophthalmic Center, Sun Yat-sen University, Guangzhou, 510060 China

**Keywords:** Cynomolgus macaques, Healthy humans, Vascular, Optical coherence tomography angiography (OCT-A), Vessel density (VD)

## Abstract

**Background:**

The metabolic activity of retina is higher than other human tissues and is crucial to the vision. Cynomolgus macaques is widely used in ophthalmic disease research. The evaluation and comparison of macular and optic disc vascular circulation parameters between normal adult cynomolgus macaques and healthy adult humans using OCT-A can promote better use of nonhuman primate models in studies of ophthalmic vascular disease.

**Methods:**

Twelve normal adult cynomolgus macaques with a mean age of 4.91 ± 0.43 years were studied for data collection. The macula of 28 adult healthy humans (14 males and 14 females), with a mean age of 25.11 ± 6.21 years and the optic discs of 9 adult healthy humans (4 males and 5 females) with a mean age of 28.56 ± 6.78 years were measured. The vessel density (VD) was measured using an RTVue XR with AngioVue. The scan sizes of the macular and optic discs were 3 × 3 mm and 4.5 × 4.5 mm, respectively.

**Results:**

OCT-A can image the superficial and deep capillary plexuses and radial peripapillary capillary network. In RPC layer of the optic disc, the VD in the nasal quadrant was lower than the VD in the inferior temporal quadrant. Similarities and significant differences in VD between healthy humans and cynomolgus macaques were obtained using OCT-A.

**Conclusions:**

This study provides normal vascular parameters for adult cynomolgus macaques using OCT-A to help establish an optical parameter database for cynomolgus macaques and compare VD between healthy humans and cynomolgus macaques to promote choroid-retinopathy research.

**Trial registration:**

Current Controlled Trials NCT03692169, retrospectively registered on 26 sept 2018.

## Background

The retina plays a vital role in vision. The metabolic activity of the retina is higher than other human tissues [1, 2]; therefore, the vascular circulation of the retina is complex. A variety of techniques have been used to measure retinal perfusion. Fundus fluorescence angiography (FFA) is routinely used to evaluate retinal vascular retinopathy [3] as it can analyse the choroid-retinal vasculature and show vascular leakage and neovascularization [4]. However, this method has limitations, such as the intravenous injection of contrast agents that can lead to some side effects [5, 6]. Additionally, further details of the deeper capillary plexus are unable to be extracted from FFA images due to limited depth perception. In contrast, optical coherence tomography angiography (OCT-A) is a novel imaging method [7–9]that detects the flow of blood via intrinsic signals without requiring an intravenous agent. OCT-A visualizes and divides the retina into various layers, clearly showing the capillary vessels in each layer. The ability of OCT-A to quantify the blood flow and vascular density of the retinal choroidal circulation is essential to the study of ophthalmology worldwide.

Due to the close similarity to humans in terms of optical structure and function, nonhuman primates such as cynomolgus macaques play a crucial role in ophthalmic disease research [10–13]. The FFA and OCT-A vascular parameters for normal humans have been previously reported. [1, 14, 15] Researchers have reported flow perfusion parameters for normal rhesus monkeys [16] and chorioretinal structure and stratification of cynomolgus macaques and rhesus monkeys by SD-OCT. [17, 18] However, very little literature is available on the vascular density parameters for the macular and optic disc of ocularly normal adult cynomolgus macaques, researchers have not clearly determined how OCT-A may be used for disease management, particularly in animal models, and the comparison of VD between healthy humans and cynomolgus macaques using OCT-A has not been conducted. Our study aimed to evaluate macular vascular circulation parameters in normal adult cynomolgus macaques using OCT-A. For a better use of nonhuman primate models in studies of ophthalmic disease, we compared the vascular parameters between healthy humans and cynomolgus macaques.

## Methods

### Animals

Data were collected from the left eyes of 12 normal adult male cynomolgus macaques with a mean age of 4.91 ± 0.43 years and a mean weight of 4.95 ± 0.84 kg. All the animals were purchased from GuangZhou Blooming-Spring Biological Technology Development Co., Ltd. (Guangzhou, China), and the animal production licence number of this company was SCXK(YUE)2014–0027. Before starting the experiments, all animals were determined to be ocularly normal and healthy. After these experiments, the animals continued to receive good care and will be used in subsequent studies.

### Preparation of animals

Animals were anaesthetized with an intramuscular injection (i.m.) of Zoletil™ 50 (VIRBAC S.A., France), which is a combination of tiletamine-zolazepam (4 mg/kg). Anaesthesia was maintained during examinations by intravenously injecting supplemental doses of Zoletil™ 50 (1/3 of the initial dose) as needed. Body temperature was maintained at 37 °C using a water circulating heating pad. Pupils were fully dilated to approximately 9 mm in diameter with tropicamide phenylephrine eye drops (0.5%, Santen pharmaceutical Co.,Ltd., Japan). Sodium hyaluronate eye drops (0.3%, Santen pharmaceutical Co.,Ltd., Japan) were used to maintain corneal moisture. A restraining device was used to maintain stable positioning of the animal’s eyes and head. The eyelids were opened with a lid speculum.

### Healthy humans

Healthy humans were recruited prospectively from the Zhongshan Ophthalmic Center, Sun Yat-Sen University, Guangzhou, Guangdong between December 2017 and December 2018. The macular of 28 healthy humans (14 males and 14 females) with a mean age of 25.11 ± 6.21 years and the optic discs of 9 healthy humans (4 males and 5 females) with a mean age of 28.56 ± 6.78 years were measured. The Ethics Committee of the Zhongshan Ophthalmic Center, Sun Yat-Sen University, Guangzhou, Guangdong approved the research protocol, which followed the recommendations of the Declaration of Helsinki. Written informed consent was obtained from each patient before any examinations were performed. Each participant’s medical history and records were carefully reviewed for retinal and cardiovascular diseases. Only participants satisfying the inclusion and exclusion criteria were included.

### Data acquisition

The left eyes of the animals and healthy humans were scanned using RTVue XR with AngioVue (software version 2018.0.0.18; Optovue, Inc., Fremont, CA, USA). The scan size was 3 × 3 mm for the macular disc and 4.5 × 4.5 mm for the optic disc.

Vessel density (VD) is defined as the percent area occupied by blood vessels. AngioVue automatically measures the VD of the scanned area of the macular as follows: the superficial layer [upper line: ILM(internal limiting membrane)with a 0 μm offset; lower line: IPL(inner plexiform layer)with a − 9 μm offset]; the deep retinal layer [upper line: IPL with a − 9 μm offset; lower line: OPL (outer plexiform layer)with a 9 μm offset]. The scanned area used to determine the VD in the optic disc was divided into several sections and the AngioVue automatically measures the VD of the radial peripapillary capillary (RPC) [upper line: ILM with a 0 μm offset; lower line: NFL (nerve fiber layer) with a 0 μm offset]. A single trained researcher acquired all images. Two experienced ophthalmology professors verified the segmentation accuracy.

AngioAnalytics flow density map software automatically evaluates the VD of the scanned area. In the macular area, the inner and outer rings with diameters of 1 and 3 mm, respectively, centred on the fovea were scanned and divided into the following six sections: fovea, parafovea, temporal (T), superior (S), nasal (N) and inferior (I), where the fovea centre was automatically determined from the relevant OCT data. In the optic disc area, a ring with a width of 0.75 mm centred on the disc was scanned and automatically divided into seven sections as follows: inside disc (ID), nasal (N), inferior nasal (IN), inferior temporal (IT), superior temporal (ST), superior nasal (SN) and temporal (T).

### Statistical analysis

The test images were loaded into Photoshop CS6. IBM SPSS statistics version 19.0 (SPSS, Inc. Chicago, USA) was used to analyse the data. The measurements are presented as means standard deviations (SD). Independent sample t-test for comparison of humans and cynomolgus macaques. The comparisons of VD in different layers and different regions of each layer were analysed using the Bonferroni analysis. The level of significance adopted in the present study was *P* < 0.05.

## Results

### VD of the macular region

**Fig. 1:**
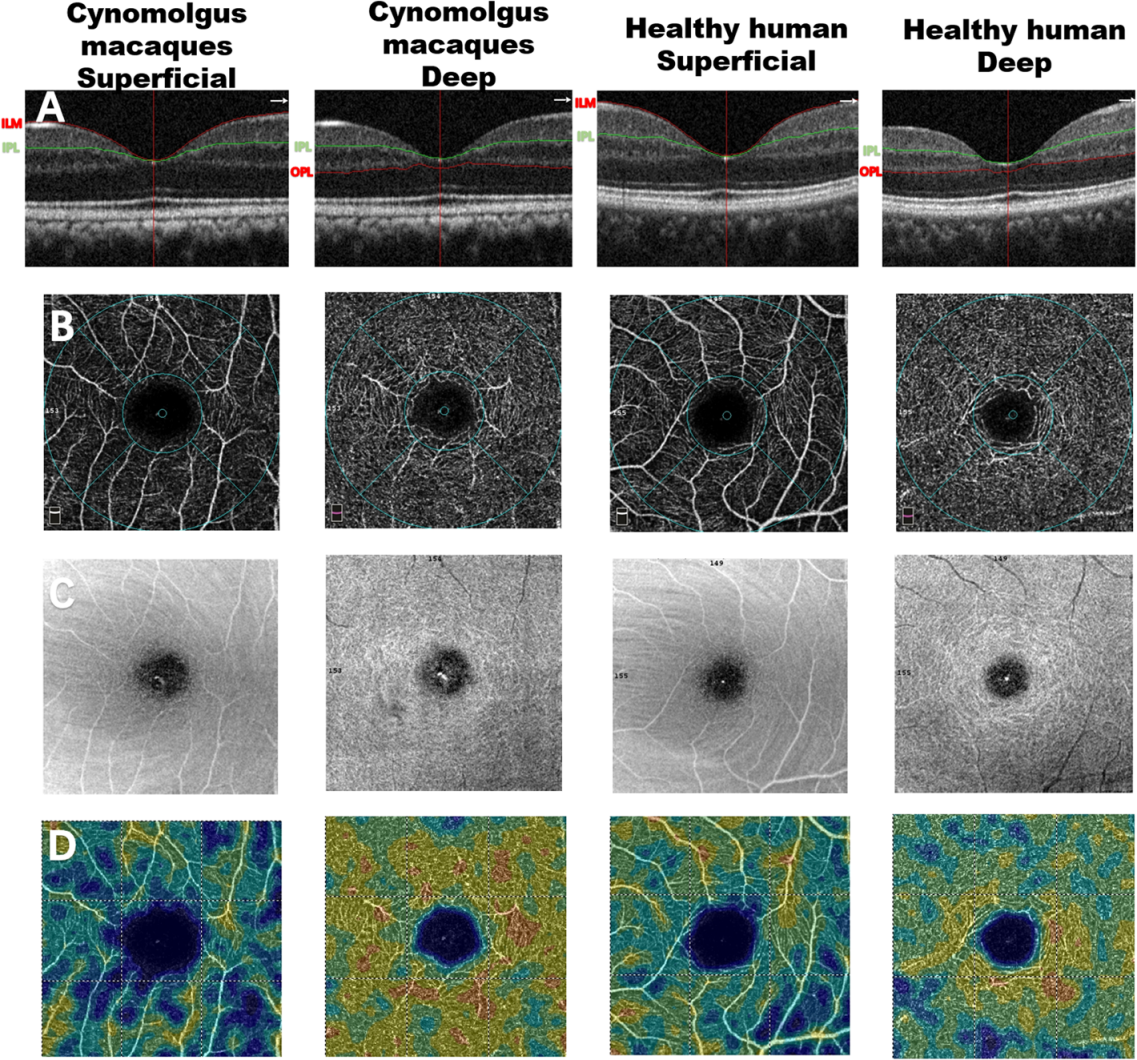
The OCT-A images of cynomolgus macaques and healthy humans in macular. **a** Retinal layer (**b**) Ring centering on macula with foveal ring (1 mm), four quadrants (superior, inferior, temporal, and nasal, 3 mm). (**c**) The En-face (**d**) Normal virtual colored macular vascular density

**Fig. 2:**
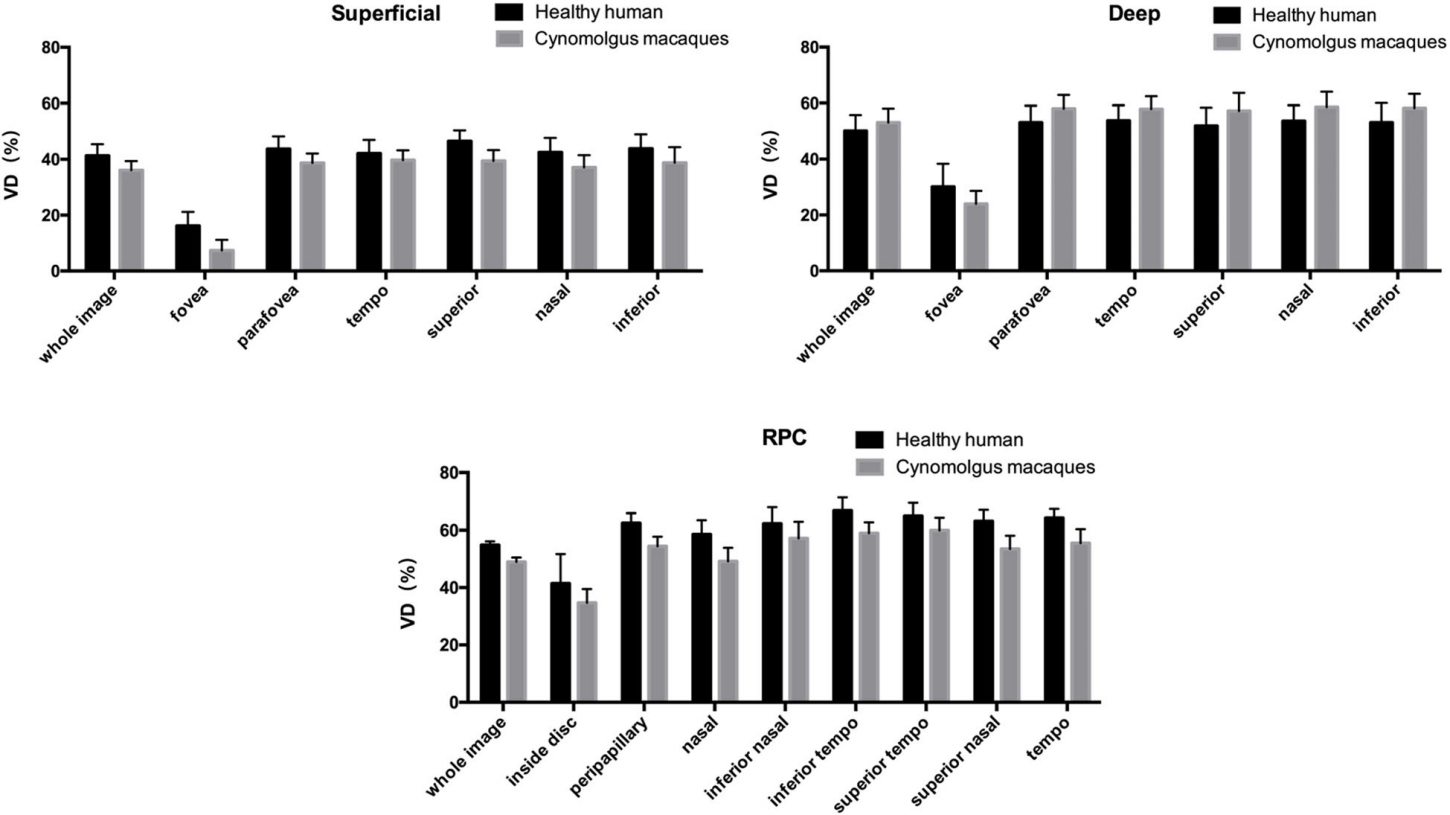
Histogram of vessel density (VD) in macula and optic disc of cynomolgus macaques and healthy humans

Figure 1 shows the two levels of choroid-retinal capillary network images in cynomolgus macaques and healthy humans obtained using OCT-A, including the superficial layer, deep retinal layer. In cynomolgus macaques and healthy human, a comparison of the complete en-face VD among the superficial and deep layer revealed that the deep layer had the higher VD (*P* < 0.05). The four peripapillary sections of each retinal layer did not show significant differences(P>0.05). Similarities and significant differences in the comparison of the VD of healthy humans and cynomolgus macaques were observed in areas of the two retinal layers as shown in Table 1 and Fig. 2. it is observed that VD in the healthy human retina is higher than in the cynomolgus macaque retina in the fovea area of superficial and deep layer.

**Table 1:** VD (X ± SD) of various sections in the macula of cynomolgus macaques and healthy humans

		Whole (%)	Fovea (%)	Para (%)	T (%)	S (%)	N (%)	I (%)
Superficial	Cynomolgus macaques (*n* = 12)	36.10 ± 3.24	7.38 ± 3.80	38.72 ± 3.38	39.68 ± 3.57	39.45 ± 3.88	37.08 ± 4.41	38.78 ± 5.55
	Healthy humans (*n* = 28)	41.33 ± 4.08	16.15 ± 5.02	43.73 ± 4.49	42.09 ± 4.87	46.47 ± 3.88	42.47 ± 5.22	43.84 ± 5.11
	*P value*	*0.00	*0.00	*0.00	0.13	*0.00	*0.00	*0.01
Deep	Cynomolgus macaques (*n* = 12)	53.08 ± 4.99	23.99 ± 4.65	58.02 ± 4.87	57.89 ± 4.56	57.25 ± 6.36	58.64 ± 5.41	58.21 ± 5.12
	Healthy humans (n = 28)	50.10 ± 5.67	30.13 ± 8.25	53.11 ± 5.99	53.78 ± 5.44	51.90 ± 6.54	53.62 ± 5.60	53.12 ± 6.90
	*P value*	0.12	*0.02	*0.02	*0.03	*0.02	*0.01	*0.03

*Whole en-face (Whole), fovea, parafovea (Para), temporal (T), superior (S), nasal (N), inferior (I)*

*means P<0.05

### VD of the optic disc and peripapillary region

**Fig. 3:**
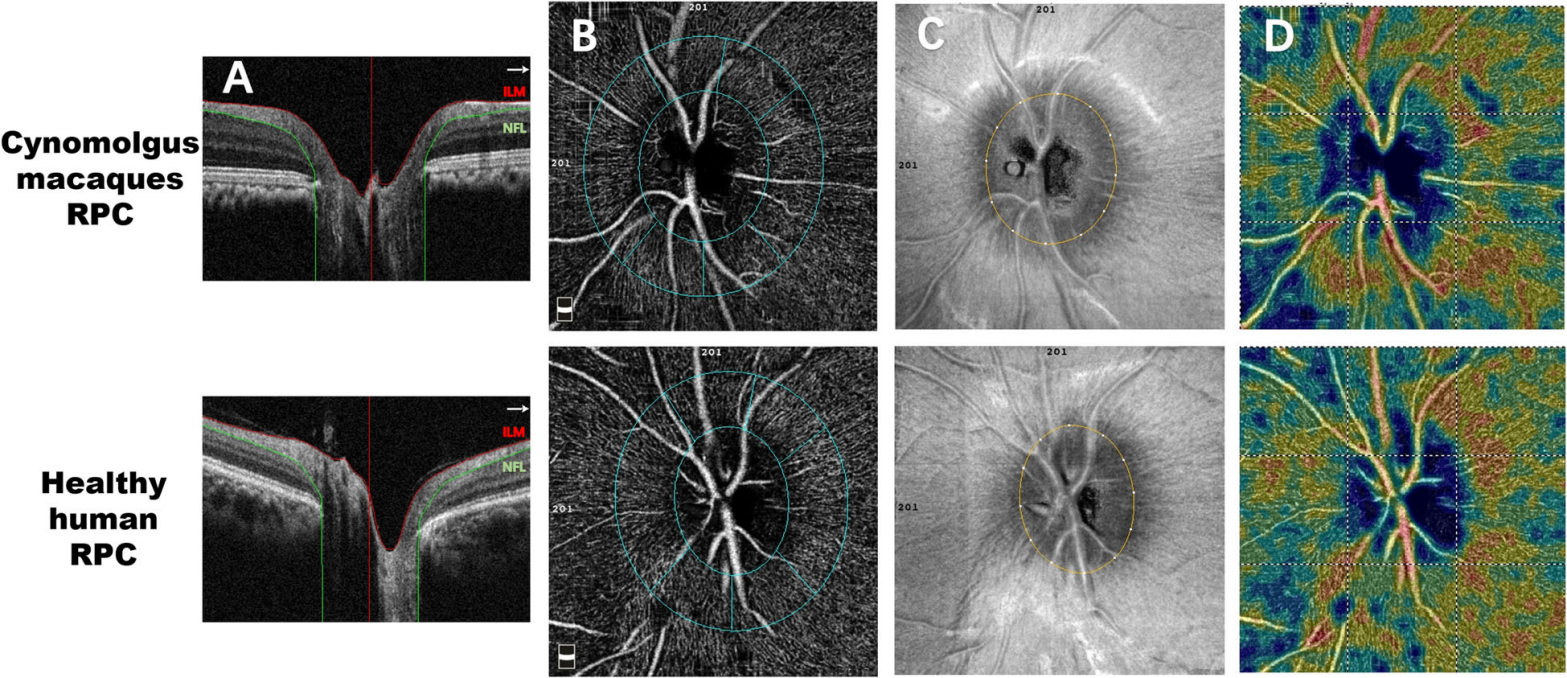
The OCT-A images of cynomolgus macaques and healthy humans in optic disc. Description: (**a**) Retinal layer (**b**) A ring with width of 0.75 mm, centering on the disc, automatically divided the scanned area into seven sections. **c** The En-face (**d**) Normal virtual colored vascular density

Figure 3 shows the RPC layer of choroid-retinal capillary network images in cynomolgus macaques and healthy humans obtained using OCT-A. In Table 2, a comparison of the measurements of RPC layer of the cynomolgus macaque retina revealed that the mean VDs of the entire en-face, ID region and peripapillary region were different (*P* < 0.05), the VD of the ID region was lower. Regarding the six peripapillary regions of the retinal layer, the VD was lower in the N quadrant than in the IT and ST quadrants (P < 0.05). Figure 2 and Table 2 show the VD of RPC layer of choroid-retinal capillary network in healthy humans obtained using OCT-A. VDs in whole area is similar in the two species. What’s more, VDs of various partitioned area were higher in healthy humans (P < 0.05).

**Table 2:** VD (X ± SD) of various sections in the optic disc of cynomolgus macaques and healthy humans

RPC	Whole (%)	ID (%)	Peri (%)	N (%)	IN (%)	IT (%)	ST (%)	SN (%)	T (%)
Cynomolgus macaques (*n* = 12)	48.96 ± 1.52	34.72 ± 4.81	54.46 ± 3.30	49.20 ± 4.69	57.17 ± 5.78	58.95 ± 3.79	59.95 ± 4.40	53.49 ± 4.62	55.53 ± 4.81
Healthy humans (*n* = 9)	54.83 ± 1.27	41.46 ± 10.22	62.51 ± 3.37	58.54 ± 4.93	62.31 ± 5.62	66.78 ± 4.60	64.94 ± 4.59	63.17 ± 3.87	64.32 ± 3.07
*P value*	1.47	0.06	*0.00	*0.00	0.06	*0.00	*0.02	*0.01	*0.00

Whole en-face (Whole), peripapillary (Peri), inside disc (ID), nasal (N), inferior nasal (IN), inferior temporal (IT), superior temporal (ST), superior nasal (SN), temporal (T). * means P<0.05

## Discussion

OCT-A is a novel imaging method [6, 19]. OCT-A was developed as an extension of OCT that clearly shows the vascular circulation in the retina and choroid [20, 21]. FFA is a conventional, qualitative technology used to diagnose retinal vascular diseases, such as diabetic retinopathy (DR) and central retinal vein occlusion (CRVO) [22, 23]. Using a special intravenous dye, FFA is one of the most valuable methods for evaluating the retinal vasculature. FFA records the filling process of the choroid-retinal vasculature and has a wide field of view.

Unlike FFA, OCT-A detects blood flow via an intrinsic signal without the use of any intravenous agents, thereby avoiding any serious side effects arising from fluorescent dyes. OCT-A saves time with quick scanning speeds. FFA unquestionably provides two-dimensional (2D) images of vascular circulation [3], but with a limited depth perception and detailed investigation of vessels [4]. The development of OCT-A solves these problems. OCT-A visualizes blood flow and divides the retina into various layers, clearly showing capillary vessels in each layer, not just transparent vessels. Therefore, deeper capillary plexuses, which are often mistaken for background choroid fluorescence in FFA, have been observed using OCT-A [4, 24]. Interestingly, the OCT-A image in which the RPC network is readily visible and the fluorescein angiographic image of the same region in our study showed considerable similarities with research by *Richard F* in humans [4]. No previous study has shown the RPC network using fluorescein angiography [25].

Researchers can quantitatively analyse vessel parameters using high-resolution imaging with OCT-A. Therefore, in the present study, we further compared the VD of the macular, optic disc and surrounding regions using OCT-A. two levels of choroid-retinal capillary networks are present in the macular of cynomolgus macaques, including the superficial layer, the deep retinal layer. Because the VD of choroid capillary can be disturbed by the inner vascular network, so we only compare the VD of superficial and deep retinal layer and find out that the deep layer had the higher VD in the two groups. These results are consistent with *Florence Coscas*’ research in humans [1]. One possible explanation for this finding is that the deep layer is formed by a homogenous capillary vortex [26], whereas the superficial layer is formed by transverse capillaries alone. Additionally, the superficial layer may artificially influence the VD assessment of the DCP [1]. The lowest VD is observed in the foveal area than in other sections of the retinal layer, probably due to the foveal avascular zone (FAZ) [21].

The VD of the peripapillary region was much higher in the RPC layer. In RPC layer of optic disc, the VD in the N quadrant was lower than the IT quadrant. Overall, OCT-A provides better images of the RPC network. The blood supply associated with early optic disc lesions in patients with glaucoma is derived from the microcirculation of the posterior ciliary artery; hence, an observation of the RPC layer is beneficial in the early diagnosis of glaucoma [27].

Despite the novelty of OCT-A, particularly in assessing different diseases [28–32], researchers have not clearly determined how OCT-A may be used for disease management, particularly in animal models. Currently, nonhuman primates, particularly cynomolgus macaques, play a very important role in the research of ophthalmic diseases due to their similarities to humans in terms of the optical structure and function [8–11]. Therefore, a better understanding of novel OCT-A parameters and a comparison of differences between healthy humans and cynomolgus macaques will help establish these techniques, particularly OCT-A, as methods for the diagnosis of diseases in animal models.

We further compared the VD of normal humans and cynomolgus macaques in the area of the RPC network and the layers of the superficial and deep retina, and all of the data showed some similarities and significant differences between two groups. In macular, the VDs of the superficial and deep layer are different in both cynomolgus macaques and healthy humans. In the fovea area of superficial and deep layer, VD of healthy human is much higher. Significant differences in the RPC layer of the optic disc were observed between the two groups, healthy humans present higher VDs in various sections. Differences between two groups of VD are obvious in the macular and optic discs. These meaningful similarities and differences should take into account in the animal researches about human optical diseases, especially vascular diseases in macular and optic disc, such as glaucoma.

## Conclusions

In conclusion, we evaluated macular vascular circulation parameters in normal adult cynomolgus macaques using OCT-A and analysed the VD parameters of the choroid-retinal vasculature and compared the vascular parameters between healthy humans and cynomolgus macaques. Obviously, the retinal structure of cynomolgus macaques was very similar to healthy humans; thus, we can use this animal model to better study the development of human optical diseases. However, some differences in the VD were observed between the two groups, indicating that when we use animal models to study optical diseases, we should also consider the functional differences between animals and humans and take these into account in the animal researches about human optical diseases, especially vascular diseases in macular and optic disc. Overall, our research provides the normal vascular parameters of cynomolgus macaques and healthy humans, promoting the establishment of an eye parameter database for nonhuman primates and animal models of ophthalmic diseases. However, our study has certain limitations that must be addressed. For example, we used cynomolgus macaques as the experimental animal and we did not clearly determine whether these results are applicable to other species. Our future goal is to conduct an extensive study across different ages, genders and species, comparing the similarities and differences in OCT-A parameters between healthy humans and nonhuman species to facilitate the research of ophthalmic vascular diseases.

## Data Availability

The datasets used and/or analysed during the current study are available from the corresponding authors upon reasonable request.

## Abbreviations

FFA: Fundus fluorescence angiography
ILM: Internal limiting membrane
IPL: Inner plexiform layer
NFL: Nerve fiber layer
OCT-A: Optical coherence tomography angiography
OPL: Outer plexiform layer
RPC: (Radial peripapillary capillary)
VD: Vessel density
